# Monkeypox Transmission and Pathogenesis in Prairie Dogs

**DOI:** 10.3201/eid1003.030878

**Published:** 2004-03

**Authors:** Jeannette Guarner, Bill J. Johnson, Christopher D. Paddock, Wun-Ju Shieh, Cynthia S. Goldsmith, Mary G. Reynolds, Inger K. Damon, Russell L. Regnery, Sherif R. Zaki, the Veterinary Monkeypox Virus Working Group

**Affiliations:** * Centers for Disease Control and Prevention, Atlanta, Georgia, USA; † Oklahoma Animal Disease Diagnostic Laboratory, Oklahoma State University, Stillwater, Oklahoma, USA

**Keywords:** monkeypox virus, transmission, prairie dog, pathology, immunohistochemistry, electron microscopy, PCR

## Abstract

During May and June 2003, the first cluster of human monkeypox cases in the United States was reported. Most patients with this febrile vesicular rash illness presumably acquired the infection from prairie dogs. Monkeypox virus was demonstrated by using polymerase chain reaction in two prairie dogs in which pathologic studies showed necrotizing bronchopneumonia, conjunctivitis, and tongue ulceration. Immunohistochemical assays for orthopoxviruses demonstrated abundant viral antigens in surface epithelial cells of lesions in conjunctiva and tongue, with less amounts in adjacent macrophages, fibroblasts, and connective tissues. Viral antigens in the lung were abundant in bronchial epithelial cells, macrophages, and fibroblasts. Virus isolation and electron microscopy demonstrated active viral replication in lungs and tongue. These findings indicate that both respiratory and direct mucocutaneous exposures are potentially important routes of transmission of monkeypox virus between rodents and to humans. Prairie dogs offer insights into transmission, pathogenesis, and new vaccine and treatment trials because they are susceptible to severe monkeypox infection.

During May and June 2003, the first cluster of human monkeypox cases in the United States was reported (*1*–*4*). Most human case-patients with this febrile vesicular rash illness were believed to have acquired the infection from prairie dogs (*Cynomys* spp.) that became ill after contact with various exotic African rodents (*Funiscuirus* spp., *Heliosciurus* spp., *Cricetomys* spp., *Atherurus* spp., *Graphiurus* spp., and *Hybomys* spp.) shipped from Ghana to the United States in April 2003 (*1*,*2*). Some African rodents from this shipment became ill and died shortly after arriving in the United States. Culture and polymerase chain reaction (PCR) demonstrated monkeypox virus in two rope squirrels (*Funiscuirus* spp.), one Gambian rat (*Cricetomys* sp.), and three dormice (*Graphiurus* spp.) (*2*). The two prairie dogs described in this report came from the same wholesale pet store where other monkeypox virus–infected rodents were housed.

In areas of Africa where monkeypox infections in humans have been documented previously, serologic surveys of wild animals have suggested that infection with monkeypox virus occurs in several species of African rodents (*5*). The virus has been isolated from skin lesions on a rope squirrel from Zaire (*6*). However, pathologic studies of naturally acquired monkeypox virus infections in animals have not been reported. Here we present pathologic, immunohistochemical (IHC), electron microscopy (EM), and molecular findings in two monkeypox virus–infected prairie dogs associated with the recent outbreak of the disease in humans in the United States. These results help elucidate the pathogenesis of naturally occurring monkeypox virus infections in mammals and shed light on possible routes of viral transmission between rodents and to humans during this outbreak.

## Materials and Methods

The two prairie dogs from whom data are presented here came from a group of approximately 200 prairie dogs that were housed at a wholesale pet store with multiple species of exotic African rodents. About 110 prairie dogs were sold before 15 reportedly became ill. Of the 15 ill prairie dogs, 10 died rapidly, and 5 exhibited anorexia, wasting, sneezing, coughing, swollen eyelids, and ocular discharge. Initially, tularemia was suspected clinically, and two of the ill prairie dogs were euthanized for pathologic confirmation. The remaining prairie dogs were destroyed (*3*,*4*).

### Culture and Molecular Analysis

Fresh lung tissue specimens were evaluated for the presence of viable infectious virus by injecting them into BSC-40 tissue culture and observing them daily for typical cytopathic effect. Fresh, unfixed tissues from lung were examined for specific signatures of monkeypox virus by using PCR. Samples were initially evaluated by single-gene PCR, followed by restriction-endonuclease fragment length polymorphism (RFLP) identification of monkeypox-specific fragment patterns (*7*–*9*). An additional novel multiplex standard PCR assay (*10*) discriminated monkeypox from vaccinia and variola orthopoxvirus species on the basis of specific DNA polymerase gene amplicons. Real time (RT)-PCR assays included a specific monkeypox virus nucleic acid signature encoded in the envelope gene (monkeypox-B6R) and orthopoxvirus nucleic acid signatures in the DNA polymerase gene (E9L non var.). Controls included DNA from monkeypox virus, other orthopoxviruses, and no-template controls.

### Pathologic Examination

Tissues were examined grossly and microscopically. Hematoxylin- and eosin-stained slides were prepared from formalin-fixed, paraffin-embedded samples of the central nervous system, conjunctivae, tongue, salivary glands, lungs, heart, liver, gastrointestinal tract, spleen, adrenal glands, kidneys, and lymph nodes. IHC assays were performed as previously described for other infectious agents in the DAKO autostainer (Dako Corp., Carpinteria, CA) (*11*–*14*). Briefly, 3-μm sections of the tissues were deparaffinized and rehydrated. Tissue sections were then digested with 0.1 mg/mL proteinase K (Roche Diagnostics, Indianapolis, IN) in 0.6 M Tris (pH 7.5)/ 0.1% CaCl_2_ (proteinase K buffer) for 15 min and later blocked with 20% normal sheep serum in Tris-saline-tween-20. Tissue sections were incubated for 60 min with a primary antibody. Primary antibodies included three polyclonal antiorthopoxvirus antibodies (rabbit antivariola virus, mouse antivaccinia virus, and rabbit antimonkeypox virus [Centers for Disease Control and Prevention (CDC), Atlanta, GA]); in addition, a monoclonal anti-*Francisella tularensis* antibody (Naval Biodefense Program, Bethesda, MD), and a polyclonal anti–*Yersinia pestis* antibody (CDC, Fort Collins, CO). This was followed by sequential application of swine antimouse or swine antirabbit link antibody, avidin-alkaline phosphatase, and naphthol/fast red substrate (Dako Corp). Sections were then counterstained in Meyer’s hematoxylin (Fisher Scientific, Pittsburgh, PA).

Positive controls included formalin-fixed, paraffin-embedded cells infected with variola virus, vaccinia virus, and monkeypox virus. Negative controls included similar cells infected with influenza A virus and human herpesvirus 1 (herpes simplex) and 3 (varicella-zoster); animal tissue samples infected with Ebola virus, *Y. pestis*, and *F. tularensis*; human skin lesions known to have human herpesvirus 1 and 3; and human skin samples with noninfectious dermatitis caused by poison ivy or drug eruptions. Negative controls for the prairie dogs specimens consisted of sequential tissue sections incubated with normal rabbit or mouse serum.

### Electron Microscopy (EM)

Specimens for EM were excised from paraffin-embedded blocks of lung and tongue in areas that corresponded to positive IHC results. Tissues were deparaffinized for 1 h in xylene warmed to 60°C, rehydrated through a graded series of alcohols, postfixed in phosphate-buffered 2.5% glutaraldehyde and 1% osmium tetroxide, stained with 4% uranyl acetate, dehydrated through a graded series of alcohols and propylene oxide, and embedded in a mixture of Epon-substitute and Araldite. Ultrathin sections were stained with 4% uranyl acetate and Reynold's lead citrate.

## Results

Gross examination of both animals revealed yellow mucoid discharge in the eyelids. One animal had a 3- to 4-mm ulcer in the center of the tongue. The lungs showed patchy areas of red-brown consolidations involving about 50% of the pulmonary parenchyma. The livers were red with a few scattered, tanned, mottled areas. Typical orthopoxvirus sequences were revealed in lung tissue samples by use of a novel multiplex PCR assay, which detected the essential DNA polymerase gene; however, the standard single-gene PCR and RFLP analysis did not show the presence of monkeypox virus. Specific monkeypox virus sequences were also obtained with more sensitive RT-PCR assays. Viral cytopathic effects were observed at days 4 and 5 in cultures inoculated with fresh lung samples.

**Figure 1:**
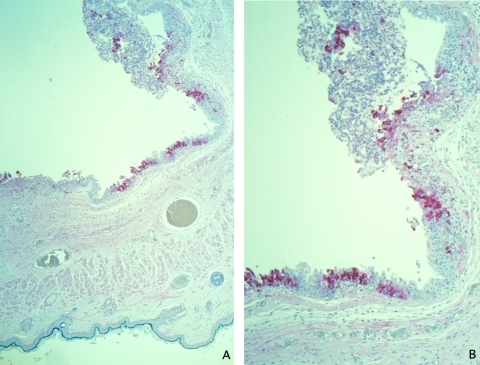
Immunohistochemical staining of a prairie dog eyelid infected with monkeypox virus, showing orthopox virus antigen staining of the cytoplasm of the epithelium of the palpebral conjunctivae (assay using anti–variola virus antibody; original magnifications: A, 12.5X; B, 25X).

Histopathologic examination of the eyelids showed a necrotic, ulcerated lesion of the palpebral conjunctiva. The ulcer bed consisted of necrotic debris and pyknotic epithelial cells. Columnar epithelial cells surrounding the ulcer were swollen and contained dense, eosinophilic, cytoplasmic granules of various sizes that suggested Guarnieri-like inclusions. The submucosa showed mixed inflammatory cell infiltrate, necrosis, and edema. Other areas of the palpebral conjunctiva and skin showed inflammatory foci in the epithelium without ulcer formation but with ballooning degeneration of epithelial cells, acantholysis, and occasional cell necrosis. Abundant orthopoxvirus antigens were detected in areas with grossly and microscopically identified lesions (Figure 1A, B) by using orthopoxvirus IHC assays. Viral antigens were present prominently in the squamous and columnar epithelium (Figure 1B) and in lesser amounts in fibroblasts and histiocytes in the ulcer bed or underlying the lesions. In epithelial cells, antigens were observed in the cytoplasm intensely staining the cytoplasmic Guarnieri-like inclusions.

**Figure 2:**
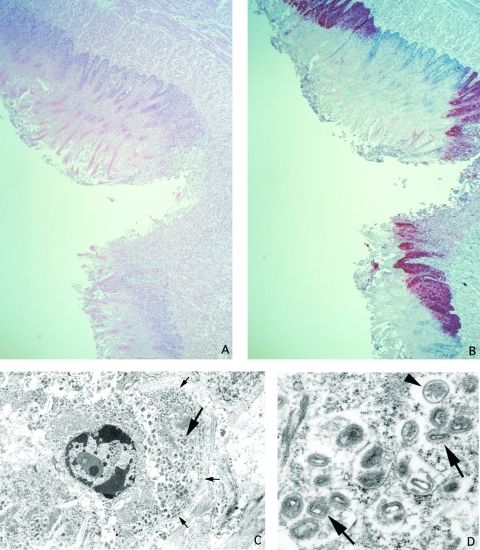
Ulcer on tongue of a prairie dog infected with monkeypox virus (A: hematoxylin and eosin stain, 12.5X original magnification). Orthopox viral antigens are abundant in the squamous epithelium, with lesser amounts in the ulcer bed (B: immunohistochemical stain using the anti-smallpox antibody, 12.5X original magnification). Tongue epithelial cell adjacent to epidermal basement membrane (small arrows) with Guarnieri-like inclusion (large arrow) (C: transmission electron microscopy, 2,400X original magnification). Higher magnification of the Guarnieri-like inclusion shows intracellular immature (arrowhead) and mature (arrows) orthopox virions. The mature virions consist of a dense core surrounded by several laminated zones and enclosed within an outer membrane. (D: transmission electron microscopy, 17,000X original magnification.)

Histopathologically, the tongue ulcer demonstrated necrosis and mixed inflammation at the ulcer bed (Figure 2A). Nonulcerated mucosa showed focal areas of lichenoid interface, mixed inflammatory infiltrate with necrosis, ballooning degeneration, and dense eosinophilic cytoplasmic granules (Guarnieri-like inclusions) in the squamous epithelium. IHC assays showed viral antigens only in lesions (Figure 2B); the antigens had a pattern similar to that described for the necrotic, ulcerated lesion of the conjunctivae. EM examination revealed abundant mature and immature poxvirus particles in the cytoplasm of epithelial cells (Figure 2C, D).

**Figure 3:**
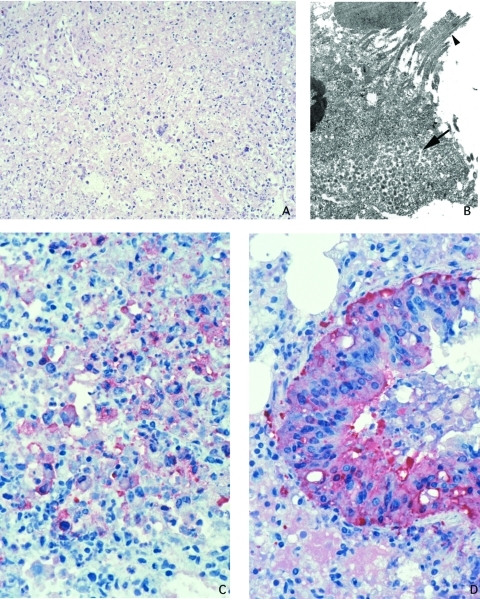
Lung of prairie dog infected with monkeypox virus, showing abundant intraalveolar mixed inflammatory infiltrate and necrosis (A: hematoxylin and eosin stain, 50X original magnification). Orthopox viral antigens are abundant in the cytoplasm of the bronchiolar epithelium (D: immunohistochemical assay anti–variola virus antibody, 100X original magnification). Macrophages, fibroblasts, and alveolar epithelial cells, as well as necrotic debris demonstrate orthopox viral antigens in pneumonic areas of the lung (C: immunohistochemical stain anti–variola virus antibody, 100X original magnification). Accumulation of intracellular mature virions (arrow) in bronchial epithelial cell (arrowhead pointing to cilia) (B: transmission electron microscopy, 2,400X original magnification).

The lungs showed concentric, coalescing bronchioalveolar pneumonia. Airways showed necrosis and mixed infiltrate of neutrophils and histiocytes in the lumen and epithelium, and the bronchial epithelium showed a reactive proliferative response (Figure 3A). Inflammation extended through the bronchilolar walls into surrounding alveoli, which demonstrated fibrinous edema, necrosis, and marked infiltrate of macrophages, some having intranuclear cytoplasmic inclusions, while others showed multinucleation. Adjacent arterioles showed reactive fibrinocellular edema in the adventitia and inflammatory infiltrate. The nonnecrotic areas of the lung demonstrated intraalveolar edema. IHC assays demonstrated abundant viral antigens in the areas with bronchioalveolar inflammation (Figure 3D). Viral antigens were observed in the cytoplasm of macrophages, bronchial epithelial cells, and fibroblasts; viral antigens were also present in the necrotic debris and interstitial connective tissue (Figure 3C). Immature and mature poxvirus particles were demonstrated inside bronchial epithelial cells by using EM (Figure 3B).

Except for mild portal inflammation in the liver and reactive hyperplasia in the spleen, no significant pathologic changes were noted in other organs. Viral antigens were not observed in other tissues, with the exception of occasional medullary and subcapsular sinusoidal histiocytes in a submandibular lymph node. No IHC evidence of *F. tularensis* and *Y. pestis* was observed in the tissues.

## Discussion

During the 2003 outbreak of human monkeypox in the United States, a shipment of African rodents that contained Gambian rats and dormice is thought to have resulted in secondary infection of prairie dogs (*1*–*4*). Exposure to infected prairie dogs resulted in 37 human infections involving exotic pet dealers, pet owners, and veterinary care workers in the United States (*1*–*3*). The mode of transmission of the monkeypox virus between infected animals and humans is not clearly defined, partly because histopathologic and immunohistochemical studies of animals with naturally acquired infection have not been published. The prairie dogs in this study demonstrated abundant viral antigens and mature poxvirus particles in the tongue and conjunctival lesions; hence, direct contact with saliva or exudates from these lesions could have inoculated monkeypox virus to skin or mucous membranes of other hosts. In addition, the lungs demonstrated abundant replicating monkeypox virus in the bronchi and lung parenchyma; thus, transmission to other rodents and humans may have occurred when the infected animal coughed and dispersed infective droplets. Furthermore, the pneumonic process in these prairie dogs suggests a respiratory route of infection between rodents. Thus, the pathologic study of severely ill prairie dogs in this outbreak provided evidence that direct mucocutaneous contact and respiratory routes played a role in transmission, as has been suggested in African outbreaks of human disease (*15*–*19*).

Prairie dogs may be an excellent animal model for the further study of monkeypox infections because they are small, plentiful, and susceptible to severe monkeypox virus disease. In naturally or experimentally infected animals, a spectrum of clinical illness will develop; for example, of the nonhuman species that naturally acquire monkeypox virus infections, skin lesions have only been observed in some African primate species and rope squirrels (*Funiscuirus* spp.) (*5*,*20*). The pathologic features observed in prairie dogs, including a necrotizing bronchopneumonia, have been described in *Cynomolgus* monkeys infected experimentally by inhalation of monkeypox virus (*21*). In these animals, the lower respiratory epithelium was the target for primary replication of virus. Monocytes carried the virus to lymphoid tissues, where a secondary viral replication occurred and resulted in the seeding of other tissues, including skin, oral mucosa, gastrointestinal tract, and the tissues of the reproductive system. In this monkey model, secondary viral replication sites had necrotizing lesions. Necrotizing lesions with viral antigens in lymphoid tissues have been seen in other animals, including prairie dogs that fell ill and died during the U.S. outbreak (data not shown). The prairie dogs in this study did not have necrotizing lymphadenitis or splenitis, which may indicate that these rodents were euthanized relatively early in the disease course.

Monkeypox virus infected predominantly epithelial cells in conjunctivae, tongue, and bronchi. Histopathologically, infected epithelial cells showed prominent ballooning degeneration and dense, eosinophilic, cytoplasmic granules that were difficult to distinguish from keratohyalin bodies. Epithelial cells occasionally coalesced, forming syncytia, and their nuclei showed eosinophilic, ground-glass staining that must be differentiated from herpetic inclusions for diagnostic purposes. By use of IHC, the eosinophilic cytoplasmic granules seen in infected epithelial cells were proven to be viral inclusions (Guarnieri-like inclusions), and EM examination corroborated these findings. In the prairie dogs studied, orthopoxvirus antigens were also demonstrated in other cells, including macrophages and fibroblasts in areas adjacent to infected epithelial cells. Histopathologic studies of human monkeypox skin vesicular lesions showed an IHC staining pattern similar to that found in the tongue and conjunctiva of the infected prairie dogs (*1*–*4*,*22*,*23*).

The U.S. monkeypox virus outbreak demonstrated how new diseases can emerge due to facile movement of species from one location to another (including the illegal transporting of species). The investigation of human cases at the wholesale pet store that housed a variety of African rodents and the two prairie dogs studied revealed a spectrum of monkeypox-associated disease ranging from only serologic evidence of monkeypox infection to febrile vesicular rash illness (*3*). Differences in disease severity may relate to the source of exposure, transmission route (i.e., inhalational versus direct mucocutaneous contact), amounts of virus inoculated, virus strain, or host susceptibility. Epidemiologic studies of human monkeypox infections have shown that younger children and persons not vaccinated against smallpox can have severe disease and complications, which supports the importance of host susceptibility, including previous immunity (*18*,*24*,*25*). This outbreak of monkeypox virus infection in humans and nonhuman animals is an important reminder to monitor surveillance programs for febrile rash illnesses designed to detect potential bioterrorism attacks with smallpox virus, which may be beneficial for detecting emerging infections (*26*).

A variety of methods were used to diagnose and study monkeypox virus infection in these prairie dogs. IHC studies permitted demonstration of the virus in the context of histopathology. EM and culture demonstrated viral replication, while molecular studies were essential for determining the specific signatures of monkeypox virus. In the prairie dogs studied, standard PCR and RFLP did not show monkeypox DNA. However, RT-PCR detected monkeypox viral DNA since RT-PCR is more sensitive and can be used to accurately titrate up to 4–10 DNA copies (*27*).

Studying necropsied animal specimens was of great benefit during this monkeypox outbreak investigation. Research into the natural biology of monkeypox has been limited because the disease is rare in humans and no descriptions exist of naturally acquired animal infections. The pathologic findings in this study of prairie dogs can be used to better define possible transmission routes and pathogenesis of human and animal monkeypox, and such a model may help develop new vaccine and treatment strategies for orthopoxvirus infections.
